# A cross sectional study on the motivators for Asian women to attend opportunistic mammography screening in a private hospital in Malaysia: the MyMammo study

**DOI:** 10.1186/s12889-015-1892-1

**Published:** 2015-06-12

**Authors:** Norhashimah Hassan, Weang Kee Ho, Shivaani Mariapun, Soo Hwang Teo

**Affiliations:** Cancer Research Initiatives Foundation, Sime Darby Medical Centre, 1 Jalan SS12/1A, Subang Jaya, 47500 Selangor Malaysia; Breast Cancer Research Unit, University Malaya Cancer Research Institute, Faculty of Medicine, University Malaya Medical Centre, University Malaya, 50603 Kuala Lumpur, Malaysia; Department of Applied Mathematics, Faculty of Engineering, University of Nottingham Malaysia Campus, Jalan Broga, 43500 Semenyih, Selangor Malaysia

**Keywords:** Mammography, Screening, Breast Cancer, Malaysia, Asia

## Abstract

**Background:**

To date, because of limited budgets and lower incidence of breast cancer, the majority of Asian countries do not have population-based screening programmes, but instead offer opportunistic screening. However, there have been few studies which have assessed the motivators for women attending such programmes and the appropriateness of the programmes in terms of targeting women at risk.

**Methods:**

We conducted a prospective cross-sectional study of 1,619 women aged 40 to 74 years attending a subsidized opportunistic screening mammogram from October 2011 to October 2013 at a private hospital in Malaysia. Breast cancer risk was estimated using the Gail Model and two-step cluster analysis was used to examine the motivators of attending screening.

**Results:**

Although Malaysia comprises 54.5 % Malay, 24.5 % Chinese and 7.3 % Indian, the majority of women in the MyMammo Study were Chinese (70.1 %) and 99.2 % had a <2 % ten-year risk of breast cancer. The most commonly cited barriers were the perception of not being at risk and fear of painful mammography. We found that highly educated women, cited doctors, family and friends as their main motivators. Of those with only secondary school education, their main motivators were doctors.

**Conclusions:**

Taken together, our results suggest the women attending opportunistic mammography screening in Asia are at low risk of breast cancer and this poses challenges to cost-effective and equitable strategies for cancer control. We propose that to improve uptake of screening mammography, awareness programmes should target both doctors and members of the public.

## Background

Breast cancer is the most common cancer in women and accounts for ~12.7 % of all cancer deaths in Asian women [1]. Although breast cancer incidence is lower in Asia compared to that in Western countries, it is now rising rapidly in Asian countries because of longer life expectancy and dramatic changes in parity and lifestyle [1–4]. The increasing burden of breast cancer in Asian countries is exacerbated by late presentation and limited access to therapies, resulting in poorer outcomes [5–7].

In high-income Asian countries with higher breast cancer incidence, such as Singapore, Korea and Japan, organized national mammographic screening is available, but uptake varies from 20 to 57 % [8–12]. In the other Asian countries, only opportunistic screening is available [13] and uptake is generally low, ranging from <1 % in Thailand [14], to ~7 % among rural Malaysian women [15–17]. A number of reasons for poor uptake have been described in Singapore, Thailand, Malaysia and Iran, and these include cost of screening, lack of time, distance to screening facilities and fear of cancer [14, 18–20].

Faced with limited budgets and an increasing incidence of breast cancer, the governments of Asian countries are facing difficult decisions on how to offer screening equitably and cost-effectively, and in particular, how to target limited resources to women at higher risk of developing breast cancer. However, there is paucity of data on the risk profile of Asian women attending opportunistic screening and limited data on how to motivate women to come forward for screening. In this study, we sought to describe the risk profile of women attending opportunistic screening at a private hospital in Malaysia, to examine the barriers and motivators of women attending screening, and to explore whether there are subgroups of healthy women with similar self-reported motivators for attending screening in Malaysia. Findings from these analyses may inform the utility of opportunistic mammography screening and further, the functional clustering may provide insight into how screening messages should be framed for more effective promotion of screening mammography in the future.

## Methods

### Study description

The Malaysian Mammography Study [MyMammo] is a subsidized opportunistic mammogram screening programme in a private tertiary hospital located in a suburban area in Malaysia. Screening was offered to women who did not have a personal history of breast cancer and who have not had a mammogram for at least one year prior to participating in the programme. Participants were recruited using flyers and posters at the hospital, and articles in the mainstream English, Chinese and Malay media. From October 2011 to October 2013, 1,619 women were enrolled into the study. Participants had a digital mammogram, donated a blood sample for research and completed a questionnaire of information in relation to demographic characteristics, anthropometric factors, menstrual and reproductive history, family history of cancer and, motivators and barriers for participating in the MyMammo study. Women were excluded if they were younger than 40 years old (n = 6), older than 74 years old (n = 4), previously diagnosed with other cancer (n = 23) or symptomatic (n = 133), leaving 1,453 in the final analysis. All women provided informed consent and this study was approved by the Sime Darby Medical Centre Independent Ethics Committee.

### Estimation of breast cancer risk

The ten-year risk of breast cancer in all women were calculated at age of participation in MyMammo study using the publicly available Gail Model by including the following variables: age at menarche (<12 years, 12-13 years, ≥14 years), age at first live birth (<20 years, 20-24 years, 25-29 years, ≥30 years), history of previous breast surgery and the number of first degree relatives affected with breast cancer. Subjects were categorized as parous if they have had at least one full term pregnancy (live or still births) and the family history of breast cancer in a first degree relative included affected mothers and sisters. For calculations of population risk to breast cancer, the population risk of the Malaysian Chinese was based on that of the Chinese Asian-Americans while the population risk of Malaysian Malay and Indian women was based on that of other Asian-Americans [21].

### Statistical analysis

SPSS software [Version 21] was used for all data analysis. The association between mammography history and baseline characteristics was investigated using Student’s *t* test and chi-square test for continuous and categorical variables respectively. A two-step cluster analysis was used to identify sub-groups of women with similar self-reported motivators for participating in the MyMammo Study. Women with previous breast biopsy (n = 136) were excluded, leaving a total of 1,317 women for analysis. Initial analysis included all variables and thereafter, variables without significant association with mammography history were removed sequentially (starting with the least significant variables). Models with acceptable cluster quality, smallest Schwarz’s Bayesian Criterion and significant differences between socio-demographic variables and motivators were selected. Differences between clusters were determined by Kruskal-wallis test for continuous variables and chi-square test for categorical variables.

## Results

### Study cohort

**Fig. 1:**
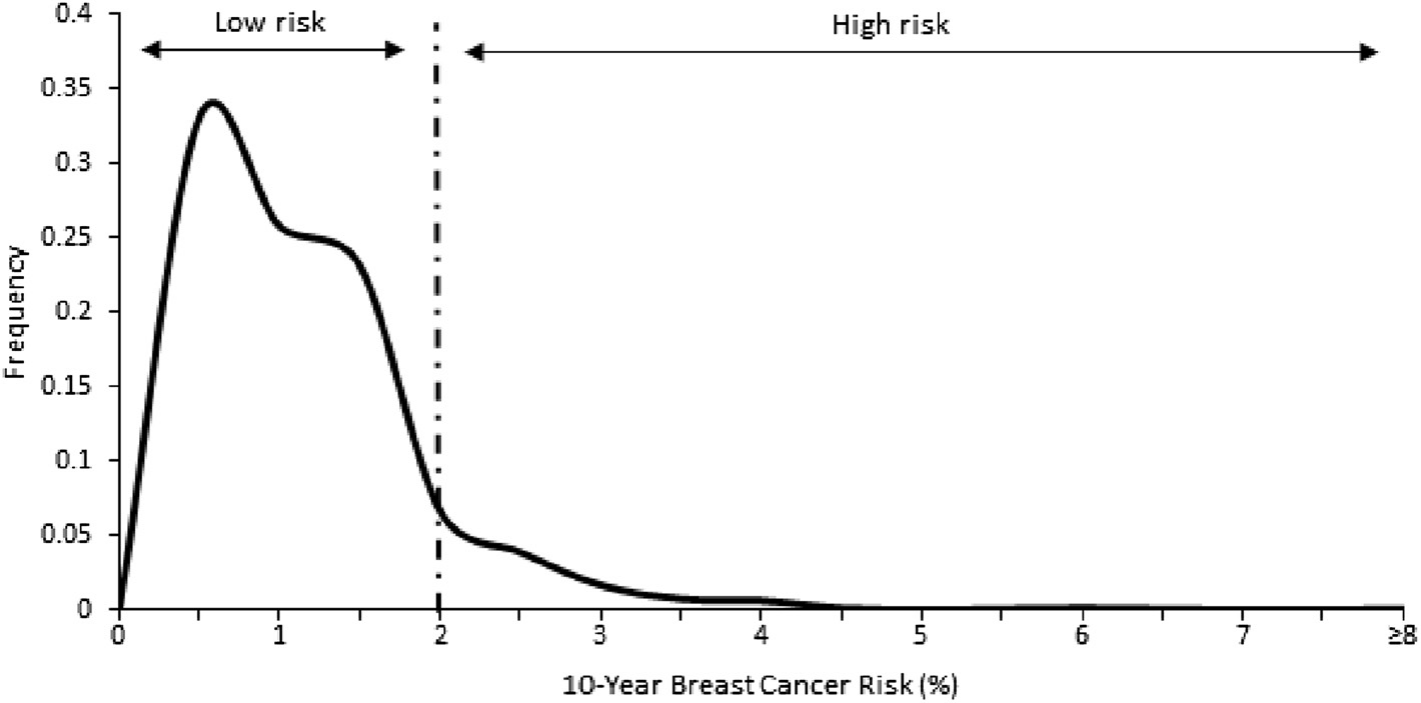
Distribution for ten-year risk invasive breast cancer in participants of the MyMammo study. Low risk is defined by having 10-year risk of less than 2 % while high risk is defined by 2 % or greater risk of developing breast cancer in the next 10 years. The majority of women (n=1415, 97.3 %) were at low risk of breast cancer and 38 out of 1453 of women (2.7 %) is predicted to be at risk of developing invasive breast cancer in the next 10 years

The MyMammo study is an opportunistic mammography screening programme established at a private hospital in the suburban area of Subang Jaya in Malaysia. The mean age was 50 years old, the majority of participants were Chinese [70.1 %], >90 % had secondary or tertiary education and 19 % were considered high income. The mean age of menarche was 13 years old and 84 % of women were parous, with a mean age of first live birth of 28 years old. Whilst 30 % of women had ever used oral contraceptives, less than 10 % had used hormone replacement therapy. Only 10 % of women report a family history of breast cancer and 9 % had previously had a breast biopsy. Twenty one percent of patients had previously had gynaecological surgery. Overall, the mean ten-year risk for women aged 40-79 was 0.77 %, ranging from 0.4 % to 14.4 %. The majority of women (n = 1453, 99.2 %) were estimated to have a <2 % risk of breast cancer, with only 0.8 % (n = 19) at 2 % risk (Fig. 1).

Women with previous mammogram were older (*p* < 0.001), tend to have higher socio-economic status (income *p* = 0.01, educational level *p* = 0.006), more likely to be menopausal (*p* < 0.001), have first live birth at an older age (*p* = 0.001), and more likely to have had hormone replacement therapy (*p* < 0.001) compared to women without previous mammogram. They also have higher proportion of positive family history of breast cancer (*p* < 0.001) and diabetic (*p* = 0.05). The remaining variables, namely ethnicity, age at menarche, smoking status and previous breast or gynaecological surgery, did not differ significantly between the two groups (Table 1).

**Table 1:** Malaysian Mammographic Study cohort description by mammography history

Variable	All women		Previous mammogram		First mammogram		*P* value
	N = 1453		N = 744		N = 709		
	N	%	N	%	N	%	
**Demographics**							
Age^1a^[mean, sd]	50.0	7.0	53.0	7.0	48.0	7.0	<0.001
Ethnicity^2a^							
Chinese	1018	70.1	530	71.2	488	68.8	0.194
Indian	223	15.4	117	15.7	106		
Malay	160		69	9.3	91	12.8	
Others	52	3.5	28	3.8	24	3.4	
Education Level^2^							
Primary	90	6.2	34	4.6	56	8.1	0.006
Secondary	728	50.1	366	49.9	362	52.3	
Tertiary	608	41.8	334	45.5	274	39.6	
*Missing*	27		10		17		
Monthly Income (RM)^2^							
Below 5 k	689	47.4	325	44.1	364	52.1	0.010
5 k to 10 k	470	32.4	260	35.3	210		
Above 10 k	277	19.1	152	20.6	125	17.9	
*Missing*	20		10		10		
**Hormonal**							
*Endogenous*							
Age at menarche^1a^[mean, sd]	13.0	1.0	13.0	1.0	13.0	1.0	0.626
*Missing*	10		5		5		
Menopausal status^2^							
Pre/perimenopausal	783	53.9	299	40.2	484	68.6	<0.001
Post-menopausal	667	45.9	445	59.8	222	31.4	
*Missing*	3		0		3		
Parity^2^							
Nulliparous	229	15.8	104		125	17.6	0.057
Parous	1224	84.2	640		584	82.4	
Age at first live births^1a^[mean, sd]	28.0	5.0	28.0	5.0	27.0	5.0	0.001
*Missing*	230		105		125		
*Exogenous*							
Oral contraceptive usage status^2^							
Never	1004	69.1	508	68.5	496	70.4	0.435
Ever used	443	30.5	234	31.5	209	29.6	
*Missing*	6		2		4		
Hormone replacement therapy							
(HRT) status^2^							
Never	1306	89.9	627	84.6	679		<0.001
Ever used	142	9.8	114	15.4	28		
*Missing*	5		3		2		
**Medical history**							
Family history of breast cancer^2a^							
No	1301	89.5	646	86.8	655	92.4	<0.001
Yes	152	10.5	98	13.2	54	7.6	
Previous breast biopsy status^2a^							
No	1305	89.8	676	91.5	629	89.6	0.224
Yes	136	9.4	63	8.5	73	10.4	
*Missing*	12		5		7		
Gynaecological surgery^2^							
No	1143	78.7	578	79.7	565	79.7	0.352
Yes	310	21.3	166	22.3	144	20.3	
Sterilisation	103	7.1	52		51	7.2	
Oopherectomy	33	2.3	21	2.8	12	1.7	
Hysterectomy	82	5.6	40	5.4	42	5.9	
Total Hysterectomy/TAHBSO	69	4.8	37		32	4.5	
Salpingectomy/salpingostomy	23	1.6	16	2.1	7		
Body Mass Index (BMI)^2^							
Underweight	48	3.3	16		32	4.5	0.030
Normal weight	812	55.9	425	57.1	387		
Overweight	576	39.6	292		284	40.0	
*Missing*	17		11		6		
Diabetes status^2^							
No	1335	91.9	673	91.1	662	93.8	0.050
Yes	110	7.6	66	8.9	44	6.2	
*Missing*	8		5		3		
Smoking status^2^							
Never	1312	90.3	671	90.2	641	90.4	0.887
Ever smoked	141	9.7	73	9.8	68	9.6	

^a^Indicates variables in the Gail Model

^1^Indicates continuous variable

^2^Indicates categorical variable

T-test was used for continuous variables and chi-square test for categorical variables

For 709 women (48.8 %) that cited the MyMammo Study as their first breast screening mammogram, 30 % (209) cited perception that they are not at risk, 20 % (139) cited fear of painful mammography, and 10 % (68) cited cost, as barriers of attending opportunistic screening.

### Cluster analysis

Whilst the association analyses revealed the differences between women who have previously attended mammogram compared with those who had not, it did not reveal whether there were subgroups of healthy women with similar self-reported motivators for attending opportunistic screening in Malaysia. To do this, we conducted a cluster analysis to determine the characteristics of women presenting for opportunistic screening, starting first with a model containing all variables in Table 1, mammography history, age at first mammogram and motivators for attending screening, and sequentially excluding variables with the least association with mammography history (Table 2). We observed that the full model and Model two generated more than three clusters with poor separation. Models 1, 3, 4 and 6, and Models 5, 7 and 8 generated two and three clusters, respectively, with fair cluster quality and similar profiles. However, only the three-cluster models show a significant difference between motivators. Although there is a slight increase in BIC value after dropping parity and menopausal status from the analysis (comparing Model 5 with Model 8), the most parsimonious model was Model 8, as it contained the least variables and all variables in the model remained significantly different between the three clusters (Table 3).

**Table 2:** Summary for two-step cluster analysis

Model	Exclusion^a^	Number of Clusters	Schwarz's Bayesian Criterion (BIC)	Cluster quality
Full model	None	4	18827.990	Poor
Model 1	Exclude smoking status	2	18959.512	Fair
Model 2	Exclude age at menarche	5	16456.869	Poor
Model 3	Exclude oral contraceptive status	2	16212.759	Fair
Model 4	Exclude Gynaecology surgery status	2	15089.163	Fair
Model 5	Exclude ethnicity	3	12973.058	Fair
Model 6	Exclude parity status	2	13416.631	Fair
Model 7	Exclude menopausal status include parity	3	13372.869	Fair
Model 8	Exclude menopausal status	3	13453.375	Fair

^a^Full model consists of all variables in Table 1, mammography history, age at first screening and motivators for attending screening. Model 1 – Model 8: sequentially excluding variables with least association with mammography history

**Table 3:** Groups of asymptomatic healthy women attending Malaysian Mammographic study using the cluster analysis (N = 989)

	Cluster 1		Cluster 2		Cluster 3		*P*-value
	N = 451		N = 262		N = 276		
	Mean	sd	Mean	sd	Mean	sd	
Age (years)^a^	52	6.0	45	5.0	51	8.0	<0.001
Age at first mammogram (years)^a^	44	6.0	45	5.0	50	7.0	<0.001
Age at first live birth (years)^a^	28	4.0	29	4.0	25	5.0	<0.001
	N	%	N	%	N	%	
Previous mammogram status^b^							
Never had a mammogram before	0	0.0	258	98.5	259	93.8	<0.001
Have had a mammogram before	451	100.0	4	1.5	17	6.2	
Education level^b^							
Primary	9	2.0	0	0.0	54	19.6	<0.001
Secondary	222	49.2	66	25.2	219	79.3	
Tertiary	220	48.8	196	74.8	3	1.1	
Monthly income (RM)^b^							
Below 5 k	180	39.9	36	13.7	236	85.5	<0.001
5 k to 10 k	164	36.4	127	48.5	37	13.4	
Above 10 k	107	23.7	99	37.8	3	1.1	
Family history of breast cancer^b^							
None	392	86.9	237	90.5	261	94.6	0.009
1 affected individuals	54	12.0	25	9.5	14	5.1	
2 affected individuals	5	1.1	0	0.0	1	0.4	
HRT Usage^b^							
Never	384	85.1	255	97.3	259	93.8	<0.001
Ever used	67	14.9	7	2.7	17	6.2	
BMI^b^							
Underweight	8	1.8	6	2.3	6	2.2	0.001
Normal weight	264	58.5	162	61.8	126	45.7	
Overweight	179	39.7	94	35.9	144	52.2	
Diabetes status^b^							
Unaffected	420	93.1	255	97.3	235	85.1	<0.001
Affected	31	6.9	7	2.7	41	14.9	
Motivators for first mammogram^b^							
Family & Friends	147	32.6	52	19.8	25	9.1	<0.001
Doctor	92	20.4	75	28.6	119	43.1	
Myself	67	14.9	16	6.1	23	8.3	
Public campaign	60	13.3	34	13.0	14	5.1	
More than 1 motivators	37	8.2	23	8.8	17	6.2	
No motivators	48	10.6	62	23.7	78	28.3	

^a^Indicates continuous variable

^b^Indicates categorical variable

The first and largest cluster (N = 451) comprised older women (mean age 52 years), all of whom have had a mammogram previously (mean age of first mammogram 44 years). Notably, women in cluster 1 had the most significant family history of breast cancer (13.1 %) and the motivators for participating in opportunistic screening were varied (family and friends, doctors, themselves and public campaign).

Cluster 2 is the smallest group (N = 262) and consists of younger participants (mean age 45 years), the majority of whom have never had a mammogram (98.5 %). They are the most highly educated (75 % have attended college or university) and have the highest monthly income (38 % earned above RM10,000 per month). Women of this cluster had the smallest proportion of overweight (35.9 %) or diabetic women (2.7 %) and the lowest use of hormone replacement therapy (2.7 %). These women were motivated to attend opportunistic screening by their doctors, family or friends.

Cluster 3 (N = 276) consists of older women (mean age 51 years), the majority of whom have never had a mammogram previously (93.8 %). Women in this cluster had the lowest socio-economic status: 98.9 % were educated at primary or secondary level, and 85.5 % reported a monthly income of lower than RM5,000. Women from this cluster had their first live birth at the youngest age (25 years), had the least significant family history of breast cancer, and the largest proportion of diabetic (14.9 %) and overweight women (52.2 %). Significantly, amongst these older women from a lower socio-economic group, the majority were motivated by their doctors to attend opportunistic screening.

Although ethnicity was excluded from cluster analysis, further examination indicated that cluster 1 had the highest proportion among Indian women (70 out of 223, 31 %) and cluster 2 the highest among Malay women (76 out of 160, 44 %).

## Discussion

The incidence of breast cancer is rising in most parts of Asia and Asian governments, particularly those from low and middle-income countries, face significant challenges in providing cost-effective and equitable screening for breast cancer. To date, the majority of low and middle-income Asian countries only offer opportunistic screening and the aspiration is that through health education and community health programmes, awareness and uptake of screening can help to ensure earlier presentation. In this study, we show that the majority of women attending opportunistic screening have an estimated <2 % risk of breast cancer in the next ten years, a threshold which is often considered where the benefits of screening outweigh the potential harms of over-diagnosis. This suggests that opportunistic screening in a private hospital in Malaysia does not target the women at high risk of breast cancer.

Previous studies using logistic regression have demonstrated a number of variables are associated with participation in mammography screening, but these studies provide limited information on how to target different groups of women [17, 18, 22]. In our study, we applied a different analytical method and suggest that functional clustering has provided insight into how screening messages should be framed for more effective promotion of mammography screening in the future. First, women with a higher risk of breast cancer on the basis of family history of breast cancer were more likely to attend screening at a younger age and they were motivated to participate in screening by various avenues. This suggests that health education focusing on the familial risk of breast cancer could be a strong motivator for Asian women to participate in screening but this approach has not hitherto been explored. Second, highly educated women could be motivated through a number of avenues including by their doctors, by family and friends or by public campaigns. Third, women in lower socio-economic classes were more likely to be motivated by their doctors to participate in screening. Taken together, this suggests that whilst highly educated women were accessible through public campaigns, women in lower socio-economic classes may not respond similarly to such campaigns. This is consistent with a systematic review of interventions to increase uptake of screening mammography among Asian women which suggests that media campaigns alone may be ineffective in increasing screening uptake [9]. In addition, our data suggests that doctors remain a significant avenue for educating women in lower socio-economic groups about screening and health education should specifically encourage primary care physicians to educate their clients about the potential benefits of screening. This is consistent with studies in Indian, Pakistani and Bangladeshi women in the UK where cultural awareness training for healthcare workers was found to increase mammography uptake [23].

The strength of the MyMammo study is the relatively large sample size and availability of data on breast cancer risk factors, which in turns enable the determination of the estimated risk of breast cancer. To date, reports on barriers and motivators among developing Asian countries have been limited to small cohorts, with limited information about breast cancer risk factors. However, the MyMammo study has a number of limitations. First, the study is conducted in a private hospital and study participants were asked to contribute a blood sample for research to determine genetic determinants of mammographic density. It is unknown whether these would have affected participation levels and the profiles of participating women. Second, as this is an opportunistic screening program, findings from this study cannot be extrapolated to the general population as it only represents a subset of women that came forward for screening.

## Conclusions

The MyMammo Study has shown that the avenues for women to become aware of and be motivated to participate in opportunistic screening is different in high-income and low socio-economic women. Findings from this study may have implications on the approach of future health education and community awareness programmes in Malaysia and other Asian countries.
